# Educating a New Generation of Doctors to Improve the Health of Populations in Low- and Middle-Income Countries

**DOI:** 10.1371/journal.pmed.1001108

**Published:** 2011-10-18

**Authors:** Francesca Celletti, Teri A. Reynolds, Anna Wright, Aaron Stoertz, Manuel Dayrit

**Affiliations:** 1 Department of Human Resources for Health, World Health Organization, Geneva, Switzerland; 2 Department of Human Resources for Health, World Health Organization, Geneva, Switzerland, and Department of Emergency Medicine and Division of Global Health, University of California San Francisco, San Francisco, California, United States of America; 3 Independent Contributor, London, United Kingdom; 4 Duke Global Health Institute, Duke University, Durham, North Carolina, United States of America

## Abstract

Francesca Celletti and colleagues from WHO argue that a transformation in the scale-up of medical education in low- and middle-income countries is needed, and detail what this might look like.

**Summary Points**

- Low- and middle-income countries need more doctors, but not simply more of the same.
- Insufficient collaboration between the health and education sectors creates a crippling mismatch between professional education and the realities of health service delivery.
- A transformative scale-up of medical education is needed to increase the capacity of health systems to respond to population needs.
- Transformative scale-up will require inter-sectoral engagement to determine how students are recruited, educated, and deployed and will assign greater value to the impact on population health outcomes among the criteria for measuring excellence.

## A Transformative Approach to Increasing Numbers and Matching Medical Education to Population Health Needs

The need to increase the number of doctors in low- and middle-income countries has been recognised as a critical health workforce issue [1],[2]. While sub-Saharan Africa has 24% of the global disease burden, it has only 3% of the world's health workers [1]. While increases in many cadres of providers—including nurses, midwives, midlevel providers, and community and other lay health workers—will be essential to mitigating the current workforce crisis, this article will specifically address the need for a transformation in physician education.

The United States has 270 medical doctors per 100,000 people, the United Kingdom 210, and Brazil 170, while Tanzania has just 2.3 and Malawi 1.1 [3]. In the 47 countries of sub-Saharan Africa, 168 schools produce only 9,000–10,000 graduates per year [4]. Increasing the number of medical graduates alone, however, will not solve the more intractable problems facing the global health workforce: the poor match between current models of medical education and evolving population health needs; insufficient alignment between the priorities and planning of the health and education sectors; imbalanced distribution that disadvantages rural and poor urban populations; and the challenges of retaining doctors in the communities where they are needed most [5],[6].

In order to transform population health outcomes, the current efforts to scale up medical education must increase not only the quantity, but also the quality and the relevance of the providers of the future. A transformative approach to medical education is needed—one that is defined by a commitment to social responsibility and insists on inter-sectoral engagement to determine how students are recruited, educated, and deployed as doctors.

In many cases today, educational institutions are isolated from national health systems and from health service delivery, limiting their ability to prepare graduates to respond to the evolving policies, epidemiology, and technologies relevant to their eventual practice sites [7]. University curricula may not accurately reflect the disease burden of the areas in which doctors are most urgently needed. Clinical training sites are most often urban tertiary centres whose practice conditions may be very unlike those graduates will ultimately face. Training physicians in isolation from other cadres may prepare them poorly for team-based practice. Finally, the failure to orient medical education to the needs of the local health care system and the most relevant models of care delivery may leave graduates unprepared to serve as advocates for improving the health care system around them. Achieving an appropriate balance between local relevance and global excellence is a challenge, though, and some have argued that placing an emphasis on social accountability in medical education can undermine the overall technical excellence of graduates. There is evidence that belies this, as graduates of some institutions committed to social accountability have been shown to secure competitive specialty training placements and demonstrate high-level academic and clinical performance [8]–[10]. Transformative scale-up of medical education should not exclude investment in centres of global excellence and world class research. Indeed, the need for specialist care is likely to increase with the improved provision of primary level care. This transformative approach simply assigns greater value to the impact on population health outcomes among the criteria for measuring excellence.

While there is increasing attention to the need for a transformation of medical education [11], there remains a paucity of published data to inform policy dialogue. Models for innovative scale-up of medical education are being implemented in a number of countries, but few outcomes have been documented. There is some literature to suggest that the articulation of a framework of generic “graduate attributes” may be an important mechanism for the development of graduate skills that transcend disciplinary content, but there is little agreement on the relevant framework for medical education [12]. It is already possible, nevertheless, to identify a number of critical areas that are in need of reform if the physician workforce of the future is to meet the needs of the 21st century. This article makes the case for multi-sectoral innovation during the scale-up of medical education—ranging from new recruitment strategies, faculty development, and curricular reform on the institutional level, to cross-sector planning and investment on the national level. Ultimately, though, innovative models must be judged on their ability to produce a new generation of doctors who are better equipped to meet the evolving health needs of the communities that they serve (Box 1).

**Box 1. The Vision for Transformative Education**

- Greater alignment between educational institutions and the systems that are responsible for health service delivery
- Country ownership of priorities and programming related to medical education, with political commitment and partnerships to facilitate reform at national, regional, and local levels
- Promotion of social accountability in medical education and of close collaboration with communities
- Doctors who are clinically competent and provide the highest quality of care
- Global excellence coupled with local relevance in medical research and education
- Vibrant and sustainable medical education institutions with dynamic curricula and supportive learning environments, including good physical infrastructure
- Faculty of outstanding quality who are motivated and can be retained

## What? Curricula Reform towards Local Relevance; Developing and Retaining Faculty in Relevant Fields

The 1910 Flexner report prompted a transformation of medical education in the US and beyond not only by highlighting inadequacies in quality and facilities, but also by making a convincing case for an approach to education that was informed by the health needs of society [13]. One hundred years later, the need for medical education to keep pace with evolving epidemiology, patient demographics, and health systems remains pertinent everywhere [14], but is particularly pressing in low- and middle-income countries. In these settings, a major transformation is needed—one that associates academic excellence with the delivery of improvements in population health outcomes.

Medical universities must teach to the local disease burden, as well as train students to practice within the care delivery models that are likely to best serve the local population health needs. The current reality is that educational institutions are not sufficiently integrated with the relevant local, regional, and national health authorities to ensure an effective alignment between medical education, research, health service delivery, and population health needs.

The current association of excellence with specialist skills, and in some cases, with training oriented to the global market, has meant that family and community-oriented medicine and public health, usually better matched to the overall epidemiological burden and needs of low- and middle-income countries, are often afforded low status and are relatively poorly paid [15]. Promoting curricula that equip graduates to address the specific epidemiology of the communities where they are deployed will be an essential part of the transformative scale-up of medical education. This includes the incorporation of community medicine and public health into curricula as compulsory rotations, with a focus on prevention and determinants of health. In addition, institutional and national funding bodies should promote research directed to national health needs and health systems.

Finally, there is increasing evidence that team-based practice with partial transfer of tasks (“taskshifting”) to non-physician providers may be the most effective means of care delivery, particularly for primary care services, in a variety of settings [16]–[18]. The form and content of medical curricula must evolve to adequately prepare physicians to practice within this model, and will likely require the incorporation of progressive educational strategies, such as interdisciplinary and inter-professional training [18]. While this article primarily addresses physician education, it emerges from the broader context of a World Health Organization initiative on medical, nursing, and midwifery education [19]. The lack of other health care workers is integral to the workforce crisis, and the scale-up of other non-physician providers will be a crucial part of any plausible solution [1],[16],[18],[19].

Further challenges relate to the need for faculty with appropriate skills and experience to teach a new generation of providers. More students will require more teachers, and yet there is an insufficient number of medical and nursing faculty to meet even current needs. Training and retaining faculty and staff is therefore of paramount importance, but a number of complex challenges must be overcome. Institutions must seek an appropriate balance between faculty teaching, service, research, and management duties to ensure that course content is relevant, that clinical skills are maintained and updated, and that career development opportunities such as research and publication are available. At the same time, institutions should develop incentive structures to ensure that teaching achievements are afforded comparable status to research and clinical work [19].

Institutions such as Walter Sisulu University in South Africa and Gezira University in Sudan have used creative approaches to faculty expansion, incorporating doctors and nurses working in district hospitals or in health clinics into the faculty body, or establishing joint appointments and affiliate positions with other institutions [19]. Developing clinical preceptor programmes can also be an effective means of expanding a mentoring pool, and can serve to bring community practitioners' understanding of local health needs into the university [20]. A number of institutions have also explored the potential of international and public–private partnerships to increase pedagogical capacity and provide opportunities for students and faculty at all partner sites.

## Who? Recruiting Trainees from Areas That Need Doctors

The new cohort of doctors will need to direct their education, research, and service activities towards addressing the priority health concerns of their communities. This may imply revision of recruitment strategies and selection criteria for students. Evidence drawn from several countries suggests that medical students recruited from marginalized communities are more likely to serve those communities for an extended period once they are qualified doctors, and community involvement in the selection of candidates may also increase engagement and retention [5],[21]. Such recruitment strategies can help identify prospective students who may be better adjusted for a lifestyle in underserved areas, more able to provide culturally sensitive and appropriate care, and more in tune with the social and economic determinants of health in the communities they serve [22]. For example, Walter Sisulu Medical School in South Africa recruits students from its surrounding communities (bridging and supplementing secondary education when necessary), whose health and social needs also guide the school's education, research, and service programmes. A total of 835 doctors have graduated, some 70% of whom still practice among the underserved rural communities of the immediate area. Others have found success abroad or as specialists, confounding skeptics who argued that the quality of the education at Walter Sisulu might prove inferior to that of more traditional medical schools [19].

## Where? Placing Training Sites Where Doctors Are Needed

In low- and middle-income countries, hospital-centred training is the norm, and both educational institutions and teaching hospitals are found predominantly in urban areas [4]. Such a concentration of opportunities in urban and specialist settings influences the types of students that are recruited and adversely affects the distribution of graduates when they enter clinical practice [5]. While the methodological quality of the evidence is limited [23], community-based learning may contribute to the responsiveness of health systems to community needs and nurture a commitment to public service among trainees. Evidence from the US shows that physicians trained through community health centres are 3.4 times more likely to work in a health centre and 2.7 times more likely to work in an underserved setting [24]. At the Jimma University Medical School in Ethiopia, combining training in community environments with an interdisciplinary approach to medical education resulted in higher-quality graduates with skills relevant to nearby populations. In Thailand, health professional trainees are recruited from rural areas, and then returned to their local communities in “hometown placement” initiatives [25]. At Gezira University in Sudan, each student is attached to a particular family for the period of their training. Student teams consult community members to identify priorities around which they develop projects and then seek funding for implementation and evaluation. In one village, Gezira students built a soap factory that made a significant contribution to tackling endemic scabies [19].

## How? Multi-Sectoral Government Policy Reforms and Planning and Alignment between Educational Institutions and Health Service Delivery

Such a re-orientation of education to population health needs has far-reaching implications and will require political commitment and engagement of multiple government sectors, of communities, and of international development partners. Currently, in most countries, high-level political commitment to medical education reform is scarce, and responsibility for medical universities lies only with the Ministry of Education. Without broad policy reforms and an inter-sectoral approach that facilitates national planning, the potential for scale-up in the production of doctors is limited, and investment in medical education is unlikely to produce maximum returns in health. For example, producing new doctors without regard for overall national human resource plans can result in a mismatch of graduates to country needs or a shortage of posts for newly qualified staff. A match between supply and demand is essential to ensure efficient and effective delivery of health services [26].

In addition, significant resource and logistics coordination will be required of government ministries and other stakeholders. Medical student numbers cannot be increased without enough well-qualified students graduating from secondary education. The need for increased infrastructure for medical education will not only require better teaching facilities, but also improvements in water, sanitation, transport, and accommodation. New doctors cannot be deployed without budgetary allocation for salaries from the ministry of finance. Scaling up medical education therefore implies strategic planning and financial investment on a long-term and multi-sectoral basis.

While the challenges are daunting, there is already evidence from nations as diverse as Brazil [19], Thailand [25], and Venezuela [27] that such innovative, multi-sectoral commitment to health professional education can reap significant longer-term savings in terms of population health outcomes and economic development. In Brazil, for example, integration of the health and education sectors at the highest level (the national constitution establishes joint responsibility over the education of health professionals to the Ministry of Education and the National Health System) has allowed for significantly improved utilization and chronic disease management. In Thailand, multi-sectoral planning facilitated rural recruitment and hometown placement initiatives that substantially increased retention in underserved areas [25]. In Venezuela, interdisciplinary coordination for educational innovation allowed very rapid scale-up of primary care services for millions of people. These initiatives have shown the potential of coordinated, multi-sectoral educational reform to produce a workforce well matched to population health needs and to increase access to primary health care, including among hard-to-reach communities.

## Conclusion

Policy discussion on the need for more doctors in low- and middle-income countries has tended to focus on the push and pull factors that influence movements within nations and across borders, and on the need for increased educational capacity [28]. These factors are indeed important and demand close analysis. However, strategies to improve retention and increase student numbers are unlikely to suffice without efforts to also address the fundamental shortcomings in current approaches to medical education.

Determining the extent of reform needed, and the means by which to achieve such a transformation, has not yet been attempted in a comprehensive and systematic manner. To this end, the work of the 2010 Commission on Education of Health Professionals for the 21st Century [11] is to be welcomed, as is the complimentary effort by the World Health Organization to produce evidence-based guidance on the transformative scale-up of medical and nursing education [19]. Now is the time to assess the available evidence, to address knowledge gaps through data collection, and to bring together existing expertise to construct a robust evidence base that can inform new policies amenable to all stakeholders.

It is essential that we begin to incorporate population health outcomes into the criteria we use to evaluate educational initiatives. Only via a more symbiotic relationship between medical education and population health will educational reform have the potential to deliver real improvements in health outcomes in the poorest regions of the globe.
